# Cardiac motion estimation from medical images: a regularisation framework applied on pairwise image registration displacement fields

**DOI:** 10.1038/s41598-020-75525-4

**Published:** 2020-10-28

**Authors:** Hadi Wiputra, Wei Xuan Chan, Yoke Yin Foo, Sheldon Ho, Choon Hwai Yap

**Affiliations:** 1 Department of Biomedical Engineering, National University of Singapore, Singapore, 117583 Singapore; 2 Department of Bioengineering, Imperial College London, London, SW7 2AZ UK

**Keywords:** Data processing, Image processing

## Abstract

Accurate cardiac motion estimation from medical images such as ultrasound is important for clinical evaluation. We present a novel regularisation layer for cardiac motion estimation that will be applied after image registration and demonstrate its effectiveness. The regularisation utilises a spatio-temporal model of motion, b-splines of Fourier, to fit to displacement fields from pairwise image registration. In the process, it enforces spatial and temporal smoothness and consistency, cyclic nature of cardiac motion, and better adherence to the stroke volume of the heart. Flexibility is further given for inclusion of any set of registration displacement fields. The approach gave high accuracy. When applied to human adult Ultrasound data from a Cardiac Motion Analysis Challenge (CMAC), the proposed method is found to have 10% lower tracking error over CMAC participants. Satisfactory cardiac motion estimation is also demonstrated on other data sets, including human fetal echocardiography, chick embryonic heart ultrasound images, and zebrafish embryonic microscope images, with the average Dice coefficient between estimation motion and manual segmentation at 0.82–0.87. The approach of performing regularisation as an add-on layer after the completion of image registration is thus a viable option for cardiac motion estimation that can still have good accuracy. Since motion estimation algorithms are complex, dividing up regularisation and registration can simplify the process and provide flexibility. Further, owing to a large variety of existing registration algorithms, such an approach that is usable on any algorithm may be useful.

## Introduction

Accurate estimations of cardiac motion from clinical scans is an important task, and can be used to aid the evaluation of cardiac function^1^, detection of dysfunction in cases such as cardiomyopathy^2^, chemotherapy toxicity^3^ and infraction^4^. It has also been used to assist with bio-mechanic studies to understand cardiac physiology based on its experience stress and strain^5,6^.

Image registration is a common way to estimate myocardial motion from clinical images^7^. With noise always present in medical images. Image registration methods includes regularisation in-built into the algorithm. Regularisation is a process of incorporation of external information to enforce smoothness into the result. It could also reduce motion estimation error by incorporating prior knowledge of the motion physics or organ physiology. It is used to enforce features found in nature such as spatio-temporal smoothness or continuity, cyclic nature of motions, and incompressibility of deformations. In the motion estimation of the heart, these features will bring greater accuracy, as the heart is known to follow these constraints^8^.

Cyclic constraint dictates that the transformation returns to its original state smoothly after one cardiac cycle, just as the myocardium should return to its initial state after its contraction. For registration techniques that march sequentially across time, the accumulation of tracking errors over many time steps will cause tracked points to drift^9^. Several solutions have been proposed in the past to solve this problem, such as using a fixed time point for reference image^10^, imposing penalty on the objective function based on the amount of drift^11^ and using a cyclic parametric model^12,13^.

The issue of temporal consistency involves ensuring that the transformation over time points can be consistent regardless of the time path taken. For example, the transformation across a large time step should be similar to the transformations of its smaller constituent time steps compounded together. Many authors mitigated this issue by using information from various registration paths, such as the combination of sequential registration and registration against a fixed reference time^14–16^. There are also other variants such as performing registration backwards in time^13^.

Regularisation is typically enforced as part of registration, but it can also be performed as an additional processing layer after completion of registration. Here, we demonstrate a framework for such an approach, and show that it is a viable option that still can have good accuracy. With a large variety of existing registration algorithms, an “add-on” regularisation framework that is usable on any algorithm may be useful. Further, since registration algorithms can be complex, dividing regularisation processes into those enforced during and after registration can simplify the registration process and add flexibility. Therefore, this study aims to improve the tracking accuracy of myocardial motion by an add-on regularisation layer of pairwise image registrations. The proposed framework is designed to enforce spatio-temporal smoothness, cyclic-nature of cardiac motion, and temporal consistency discussed previously.

In our approach, temporal consistency and cyclic constraint can be satisfied by having a spatial temporal model of the motion. Spatio-temporal model has been used previously by Metz et al.^12^ and Ledesma-Carbayo et al.^13^ as cubic b-spline. There are some examples of the use of harmonics such as with spatial and temporal cosine^17^ and Fourier series^18^. Our spatio-temporal model is a unique combination of these, using b-splines in the Fourier domain, which we dubbed as b-spline Fourier (BSF). Temporally, Fourier base functions satisfy the temporal cyclic constraint by accounting ensuring its return to the initial state. Meanwhile local support of spatial b-splines is capable of capturing local variations in tissue stiffness and deformation. In this study, we detail the definition of BSF, optimisation of its fit to the pairwise image registration, and mapping a coordinate to the b-spline Fourier reference frame. We further demonstrate the feasibility of the proposed framework by comparison of tracking accuracy against data from Cardiac motion Analysis Challenge (CMAC)^19^. Additionally, to demonstrate its versatility, we applied it to our own data set from various imaging modalities and animal models. The source code of the algorithm used is available in github as ’WeiXuanChan/motionSegmentation’. Also, data sets and results are available online in IEEE:DataPort as ’B-spline Fourier dataset’.

Understanding the methods used by CMAC participant is important to make informed comparison. Three of the four participants of the CMAC performed cardiac motion tracking from the ultrasound data: Fraunhofer MEVIS (MEVIS) from Germany, the Inria-Asclepios project (INRIA) from France, and Universitat Pompeu Fabra (UPF) from Spain. Each of the groups used their own unique algorithm, which would be described below.

INRIA utilises incompressible log-demons^20^ to register pairs of images. The log-demons model the deformation field as the temporal integration of a unit time at points in the image, subjected to a stationary velocity field. Only the exponential of the velocity fields were considered, resulting in a search within the log-domain. The exponential ensures that the obtained deformation would be diffeomorphic to guarantee an invertible deformation^21^. Pairwise registration were done between the first time point to the rest of the time points. Temporal smoothness was enforced by utilising the registration results from the previous time point as an initialisation for the registration at the next time point.

MAVIS group’s method could be classified under Fourier based method, which approximate local displacement by the phase shift in Fourier domain. The phase shift is detected using series of quadrature filters, to cause changes in the phase of the image^22^ and interpolating the filter that optimises some similarity metric. Pairwise registration were performed sequentially from one time point to the next. The displacement obtained were used as an input for the Morphons, which utilises information such as tissue elasticity and confidence values for each displacement field as prior for regularisation^23^.

UPF group’s method utilised a free form deformation based on a parametric model based on^9^. Regularisation in the form of incompressibility of the myocardium were satisfied by divergence free condition of the velocities. Additional regularisation were done by utilising the smoothness of the b-spline basis curves to describe velocity field in 3D+t. The similarity metric to be optimised were defined as the sum of mean square difference intensity from pairwise sequential registration and pairwise registration of other images against the first time point. These similarity measures provides coupling across the various time points to improve temporal consistency.

## Method overview

The overall framework is thus described as:

1. 3D pairwise registration
2. Forward marching
3. B-Splines of Fourier (BSF) initialisation
4. Consistency Correction of BSF
  (4.1) Cost function optimisation
  (4.2) Additional vector field inputs
5. B-spline Fourier mapping

**Figure 1:**
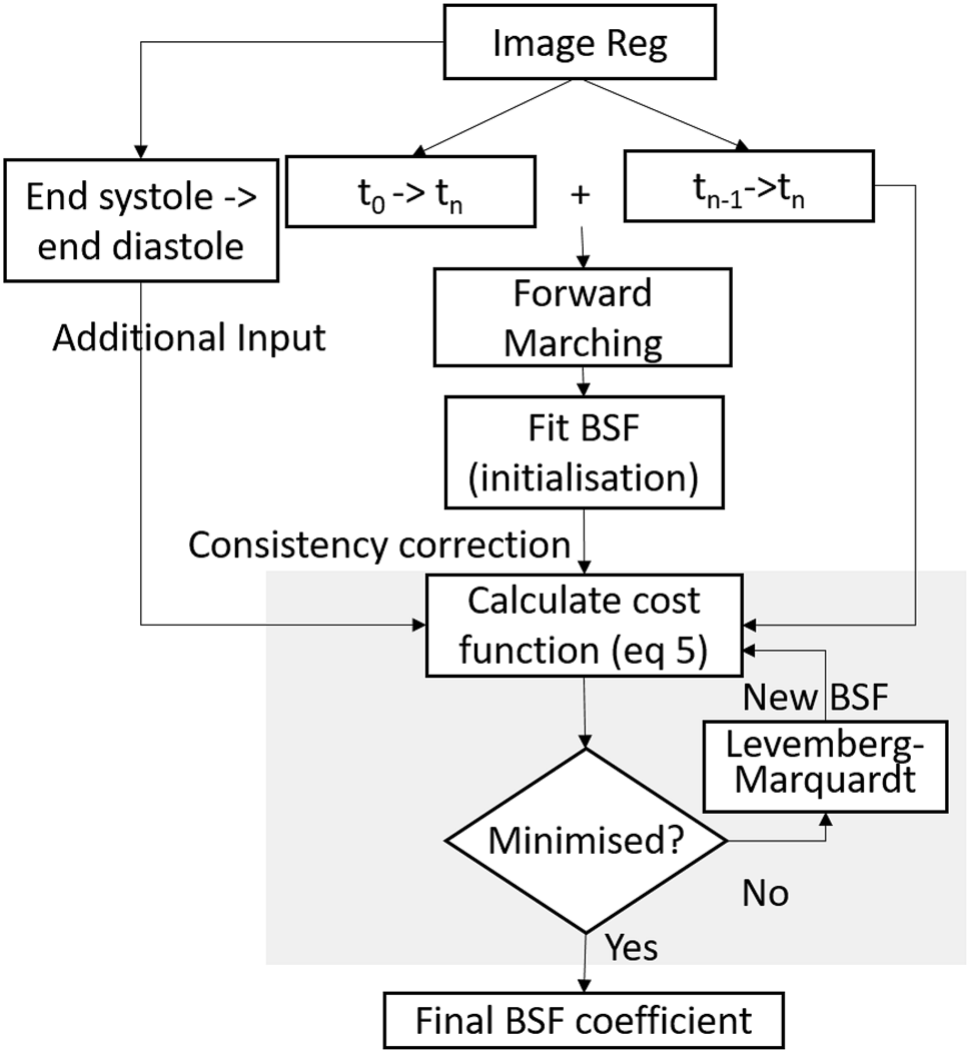
Schematic diagram of the proposed regularisation. $t_{n-1}\rightarrow t_n$ is image registration from time n-1 to time n. Forward marching accumulates image registration displacements to obtain coordinate change over time. Initialisation then sample these coordinates to fit into the b-spline Fourier model (BSF).

The proposed regularisation framework is applied onto displacement fields obtained from 3D pair-wise non-rigid image registrations in step 1. As the displacements have to be compared against coordinate ground truth, these displacement fields have to be accumulated over time, to track landmark coordinates. This process comes in “forward marching” step, which merely add displacements up without modification. It serves as baseline results to compare to improvements that can be given by our further regularisation layers. The regularisation starts when these coordinates are fitted into a cyclic mathematical model of motion (BSF model), during “initialisatiom”. The final regularisation is performed with “consistency correction” to ensure consistency among the different pairwise registrations by reducing the disparity between BSF model and all pairwise registration fields. Additional image-registration displacement fields can be used to provide fitting to constraints to improve accuracy when required. The overall framework can be represented in Fig. 1.

### Pairwise registration

Our regularisation steps can be applied to any pairwise registration algorithms, but we demonstrate its use in two algorithms. First, free form deformation algorithm based on cubic b-spline, available as python module: SimleElastix^24^. Details of the algorithm can be found in^25^. SimpleElastix obtained the optimal b-splines transformation using adaptive stochastic gradient descent on samples randomly scattered throughout the images. Mean square difference was used as the similarity metric and Transform bending energy was used as regularisation with equal relative weight between them. The b-spline control points were regularly spaced by 10 units of the largest voxel dimension spacing. Registration parameters according to default settings were currently used, and future optimisation may be possible. Second, Symmetric Log domain Diffeomorphic Demons (SLDD)^26^. Default settings were used, with Gaussian standard deviation of 3 pixels for smoothing. In both algorithms, pairwise registrations were done between each frames with the next, as well as between the first frame and the rest of available frames.

In this study, the displacement vector field obtained from image-registration is denoted as $d\vec {X}_{t\rightarrow t+dt}$, where $\vec {X}$ is the coordinate of a point in the field, *t* is the time point of the deformed image and $t+dt$ is the time point of the reference image. We denote the set of all pairwise registration displacement fields, *Reg*, such that $d\vec {X}_{t\rightarrow t+dt}\in Reg$.

### Forward marching

The forward marching step accumulates registration displacement fields over time, so that landmarks can be tracked. It serves as a baseline result to be compared to result of further regularisation layers to check for improvements. Several marching approaches are possible: Eulerian-based reference, Lagrangian-based reference, or a combination of both.

In the Eulerian approach, data from image registration between consecutive time frames is used (t → t+1). Image registration displacement vectors will always be evaluated at a fixed regular grid overlaid on the image, across all time points. This way, landmark drifting is likely to occur as error accumulates as it moves from one time point to the next.

In the Lagrangian approach, thse image registration data used is between any image and a fixed reference image (0 → t). Displacement of landmarks are calculated from the reference frame, akin to Lagrangian tracking of particles. Landmarks will return to their initial location by setting displacement to be zero after a cycle. However, the Lagrangian approach generally results in a less smooth displacement change over time since it does not take into account the state of the previous time point. Moreover, it will likely face greater difficulty in ensuring registration accuracy, as images across greater time gaps have greater differences.

A weighted average of the two approach may provide part of their individual advantages. Therefore, we test some weighted average approaches, using quadratic, Gaussian time-varying weights, and fully Lagrangian approach. Both the quadratic and the Gaussian functions model the decay of confidence on Lagrangian registration as we move further away from the Lagrangian time frame. While the different weights could affect the initialisation accuracy (differences are $<5\%$), its effect diminishes ($<0.1$%) after consistency correction (Supplementary Table 1). The different weights tested is no way exhaustive. However, we found that the accuracy post consistency correction did not changed much ($<0.1\%$). Therefore, we do not specifically recommend any approach over the others. For the purpose of illustration, the quadratic weighted average is used on all of the data sets tested. The forward marching step could then be described by the equation:

1 $$\begin{aligned} \vec {X}^{t+1}_{march}&=w(t){\times }\left(\vec {X}^t_{march}+d\vec {X}_{t\rightarrow t+1}\right)+(1-w(t)){\times }\left(\vec {X}^{0}+d\vec {X}_{0\rightarrow t+1}\right) \nonumber \\ w(t)&=\frac{(P-t)t}{(P/2)^2} \end{aligned}$$

where $\vec {X}^t_{march}$ is the coordinate in Cartesian form at time frame *t* obtained from the forward marching step. $d\vec {X}_{t\rightarrow t+1}$ and $d\vec {X}_{0\rightarrow t+1}$ are the displacement defined in Eulerian (neighbour registration) and the Lagrangian frame (registration against first frame). *P* is the period of the cardiac cycle. *w*(*t*) is quadratic such that the closer the time point is to $t=0$, $1-w(t)$ is large and decays as *t* get farther from $t=0$.

### B-splines of Fourier initialisation

#### Definition of b-splines of Fourier

The BSF model is described as follows:

2 $$\begin{aligned} \vec {X}^{t}_{BSF}=\sum _{f=0}^{N}\vec {F}_{f}{\times }cos\left( \frac{2{\pi }ft}{P}\right) +\vec {F}_{f+N+1}{\times }sin\left( \frac{2{\pi }ft}{P}\right) \end{aligned}$$

where $\vec {X}_{BSF}$ is the Cartesian coordinates of over time, *N* is the number of Fourier frequency modes used, and $\vec {F}_f$ are the Fourier coefficients, such that $\vec {F}_f \in {\mathbf {R}}^3$. The 3 element in $\vec {F}_f$ corresponds to the 3 Cartesian spatial dimensions and *f* is the frequency mode. The spatial distribution of the Fourier coefficients is described with cubic b-splines, whose control points are set up in a regularly spaced grid:

3 $$\begin{aligned} \vec {F_f}&=F_f(\vec {X}_{ref})=\sum _{l=0}^{3} \sum _{m=0}^{3} \sum _{n=0}^{3} B_{l}(o)B_{m}(p)B_{n}(q)\vec {C}_{i+l, j+m, k+n, f} \end{aligned}$$

where $\vec {X}^{ref}$ is the coordinates in the BSF reference domain. This is the domain where the b-spline control points are regularly spaced such that the the linear relationship described above is valid. *o*, *p*, *q* are defined as $(o, p, q)^T=\vec {X}_{ref}-\lfloor \vec {X}_{ref} \rfloor$. Additionally, $\vec {C}_{i,j,k,f}$ is the BSF coefficients at the control point with lattice indexes *i*, *j*, *k*, where $(i , j , k)^T=\lfloor \vec {X}_{ref} \rfloor -1$, for the Fourier frequency of *f*. $\vec {C} \in {\mathbf {R}}^3$, where the values of its 3 elements correspond to the three spatial directions. $B_{n}$ denotes the four cubic b-spline basis functions, where n={0,1,2,3}^27^.

In the initialisation step, we calculate the first estimate of $\vec {C}$ which will be further optimised during consistency correction. For clarity, all variables used as initial estimate is labeled with a superscript ’init’ (e.g. $\vec {C}^{init}$).

#### Obtaining $\vec {C}^{init}$

To calculate $\vec {C}^{init}$, we first seek the Fourier coefficients that can describe trajectories of forward marching sample points. This is achieved by seeking $\vec {F}_f$ values that fit $\vec {X}_{BSF}$ to $\vec {X}_{march}$ in the least square sense. $\vec {F}_f$ values are sought at regularly sampled point in the reference time frame, taken to be the first time frame at this stage. Since $\vec {X}_{BSF}$ is a linear function of $\vec {F}$ (Eq. (2)), the least square solution can be obtained with the Moore-Penrose inverse. We label the result as $\vec {F}^{init}$, which are samples of Fourier coefficients distributed in $\vec {X}^{ref}$.

Once the Fourier coefficients are obtained, we adopt a new reference frame, where each element in $\vec {F}^{init}$ is assigned a new corresponding coordinate location, situated at the mean of its $\vec {X}_{BSF}$ path (or the value of the 0th order term of the Fourier). This defines the BSF reference frame that lies in the centre of the motion which satisfies the zero mean displacement constrain used often in groupwise registrations^28,29^. This frame is useful in two ways: firstly, it requires minimal deformation to transform to time frames with extreme volumes so that motion estimation errors can be reduced. Secondly, having this reference frame decouples the Fourier model from any physical time point, and prevents over-reliance on information at that particular time point.

Next, scattered data interpolation with Multilevel b-spline (MLB)^27,30^ is used to calculate $\vec {C}^{init}$ from $\vec {F}^{init}$ at the control points. This is done independently for each Cartesian directions (3D). The MLB approach makes use of a hierarchy of control grid, from a course grid to a fine one, to generate a sequence b-splines whose sum approaches the value of scattered points. With this algorithm, the outputs will thus be both $\vec {C}^{init}$ values and the grid size, based on the number of levels of MLB used. higher levels allow for modelling of finer spatial fluctuations of $\vec {F}^{init}$, while lower levels smooth these fluctuations. We note that $\vec {C}^{init}$ can also be solved using Moore-Penrose inverse, since $\vec {F}_{f}$ is also a linear function of $\vec {C}^{init}$ (Eq. (3)), but this approach is less stable than MLB, especially when applied to non-regularly spaced $\vec {F}^{init}$.

### Consistency correction of B-splines of Fourier

The next step involves iterative optimisation of the fit between the BSF model and the image-registration displacement fields. Modelling $\vec {X}$ as temporal Fourier functions results in temporal smoothing and departure of $\vec {X}_{BSF}$ from $\vec {X}_{t\rightarrow t+dt}$. At the new coordinates given by $\vec {X}_{BSF}$, it will be subjected to different displacement vectors from $\vec {X}_{t\rightarrow t+dt}$, this cause inconsistency between the BSF result and the pairwise registration vectors. To solve this problem, we can fit $\vec {C}$ into sample points of $\vec {F}$ that optimises:

4 $$\begin{aligned} \forall \vec {X} \in S, find: \vec {F}^*&={{\,\mathrm{argmin}\,}}_{\vec {F}}\left( \sum \limits _{t}\sum \limits _{Reg} \frac{ {||w_{t\rightarrow t+dt}((\vec {X}^{t+dt}_{BSF}-\vec {X}^{t}_{BSF})-d\vec {X}_{t\rightarrow t+dt})||_2}^2}{2}\right) \nonumber \\&= {{\,\mathrm{argmin}\,}}_{\vec {F}} \frac{(\vec {dif})^T\vec {dif}}{2} \end{aligned}$$

where $\vec {F}^*$ is the optimised $\vec {F}$ at every points in sample *S*. *t* is time and *Reg* is set of pairwise registrations obtained. $w_{t\rightarrow t+dt}$ is the weights corresponding to each $d\vec {X}_{t\rightarrow t+dt}$, signifying the relative importance of this particular vector field. $\vec {dif}$ is the vector form of $w_{t\rightarrow t+dt}\left( \left( \vec {X}^{t+dt}_{BSF}-\vec {X}^{t}_{BSF}\right) -d\vec {X}_{t\rightarrow t+dt}\right)$. In this approach, optimisation is be performed node-by-node, and at each sample points to reduce computational time. The approximate value of $\vec {C}$ can then be obtained from $\vec {F}^*$ using the Moore–Penrose inverse to solve Eq. (3).

At every node, $\vec {F}^*$ is solved with the Levenberg–Marquardt algorithm^31^, the details of the optimisation parameters are included in supplimentary material. In essence, the consistency correction acts as a maximum likelihood approximation of the BSF parameters ($\vec {C}$) that is found close to the $\vec {C}^{init}$. We further note that the consistency correction formulation is generalised to allow inclusion of any set of pair-wise image-registration displacement fields (not limited to time-neighbouring pairs), and can thus be robustly adjusted to practical needs. In this study, only image-registration of neighbouring time points with uniform weight is used for the consistency correction step. However, additional registration inputs with uneven weight can be used when required.

#### Additional vector field inputs

Additional input of pair-wise image-registration in the consistency correction optimisation can be important in certain situations. It can, for example, constrain the consistency correction to put emphasis on images of certain time frames, such as when the heart is at the smallest and largest sizes, so that the image-tracking can calculate stroke volumes more accurately.

This additional input method are applied to our fetal and chick embryo ultrasound data. Segmentation is first performed at the end systolic and the end-diastolic image. The segmented images are binarised, filtered with the 3D Gaussian kernel with kernel size of 9 $\times$ 9 $\times$ 9 voxel, and identical standard deviation of 2 voxel in each dimensions to produce soft edges. Pair-wise image-registration is then performed between the two binarised and filtered images. The resulting vector field is given high importance during consistency correction, with a weight of 10 times that of the vector fields from image-registration of neighbouring time points, which was found to maximise the Dice coefficient result of fetal1 data. Despite the high weight, the path taken by landmarks are not constrained. Rather, it ensures that a sufficiently large displacement is taken between the time frames of the end diastolic and systolic volumes.

### B-spline Fourier mapping

In the BSF model, there exist a reference frame at which the b-spline control points grid is regularly distributed in space. We call this as the b-spline Fourier reference frame ($\vec {X}_{ref}$). It can be an arbitrary time frame (e.g first frame) or the motion-average frame. To be able to calculate the motion of a landmark over time, first, it has to be mapped to a coordinates on this reference frame where BSF is defined.

Given the coordinates of a node at an arbitrary time point, we would like to be able to calculate its corresponding coordinates at the reference frame so that the corresponding Fourier coefficients can then be obtained. The problem statement is then: given $\vec {X}^t$ and $\vec {C}$, find $\vec {X}_{ref}$ that satisfies Eq. (2), which is equivalent to finding the root of the following cost function (derived from Eq. (2)):

5 $$\begin{aligned} Cost(\vec {X}_{ref})&=\left| \left| \left[ \sum _{f=0}^{N}F_{f}(\vec {X}_{ref}){\times }cos\left( \frac{2{\pi }ft}{P}\right) + F_{f+N+1}(\vec {X}_{ref}){\times }sin\left( \frac{2{\pi }ft}{P}\right) \right] -\vec {X}^{t}\right| \right| ^2 \end{aligned}$$

where *Cost* is the Euclidean distance between current coordinate of $\vec {X}^{t}$ and the one derived from trial value of $\vec {X}_{ref}$. *Cost* were optimised with Levenberg–Marquardt with additional stochastic property. $\vec {X}_{ref}$ will descent from its coordinate at time t as the its initial value, and be updated based on formulation described in supplimentary material. Convergence is considered achieved when $Cost(\vec {X}_{ref}) < 10^{-6}$, which took less than 50 iterations in most cases.

## Studied data

Our regularisation framework is tested on four types of data sets. The first is from CMAC^19^, where 4D ultrasound images (obtained using 3D ultrasound probe) of 15 adult human hearts were obtained, together with tagged MRI data to serve as tracking ground truth. Detailed description of the acquisition is available from CMAC^19^. For each subject, 12 points on the myocardium were manually tracked in tagged MRI images by two different observers. Three of the four participants of the challenge performed cardiac motion estimation from the ultrasound data: Fraunhofer MEVIS (MEVIS) from Germany, the Inria Asclepios project (INRIA) from France, and Universitat Pompeu Fabra (UPF) from Spain, and we compare our accuracy to theirs.

In our use of data from this source, no image pre-processing is performed. During Euclidean distance error computation, time point mismatch between the ultrasound and tagged MRI data is bridged via cubic spline interpolation to obtain values at synced time points. In the ground truth landmark motion data provided, some landmarks are found to remain stationary for more than 3 consecutive time points. After contacting the CMAC author, this repetition may result from observer not noticing a change in the landmark location or simply be a manual tracking error. As caution, we thus excluded tracked landmarks that does not move for more than 3 time points. Further, if more than half of the landmarks tracked by the particular observer were excluded, the entire observer’s data for that particular subject would be excluded as well. However, we also analyze the scenario where these landmarks are included. The inclusion and exclusion of the landmarks in question, as well as the full results are listed in supplementary material (Supplementary Tables 2, 3).

Further, additional test data of our own were obtained for fetal, zebrafish and chick embryo. With 3 data for each cases, which we would refer as fetal1, fetal2, and fetal3. Also, similar labelling are used for zebrafish and chick embryo cases. We worked with broad dataset to display versatility of the proposed algorithm, and not meant to be used as direct comparison. The fetal 4D ultrasound images were obtained from 3 healthy 21 weeks old human fetuses, from the National University Hospital in Singapore. Acquisition was performed with the Spatio-Temporal Image Correlation (STIC) mode using the GE Voluson 730 ultrasound machine with the RAB 4-8L transducer (GE Healthcare Inc., Chicago, Illinois, USA), and scan data were extracted using the 4Dview software (GE Healthcare Inc., Chicago, Illinois, USA). STIC scan stitches multiple 2D images into 3D object, over various time points. The scans provided 27 volume images for 1 cardiac cycle, each of which was a stack of 27 image slices spaced 0.5 mm apart. The study protocol was approved by the Domain Specific Review Board of the National Health Group (Singapore) and written informed consent were obtained from the mother. All research was performed in accordance with relevant guidelines/regulations. No image pre-processing was done with this data set.

The third test data is 3 volumes of 4D high-frequency ultrasound images of 4.5 days old chick embryos, acquired via our previously established methods^32^. After removing the egg shell and membranes covering the embryo, a sterile polyurethane membrane was placed on the embryo to separate it from the ultrasound gel that was subsequently placed above the membrane. The vevo2100 ultrasound with the 50MHz transducer (Ultrasonix Medical Corporation, Richmond, British Columbia, Canada) was used to scan the embryonic heart at 30–40 imaging planes spaced 0.05 mm apart, using a micro-translation stage to adjust imaging location. Since the images did not have a natural contrast between tissue and blood spaces, post processing was performed, where images of multiple heart beats were ensemble averaged into one heart beat via quadratic averaging. Zero mean normalised cross correlation of every frame in one slice were calculated against every frame in the other slice to create a 2D correlation map. The temporal phase shift is detected by finding the maximum cross correlation of each frame against frames of neighbouring slice and fitting these peaks to a linear regression line. The y-axis intercept of this line is the estimated phase shift, such that these images can be synchronised and stacked to give 4D images. Finally, the contrast were further enhanced by Contrast Limited Adaptive Histogram Equalization (CLAHE)^33^.

The fourth test data was obtained from microscopy of 3 zebrafish embryonic hearts, acquired and processed like our previously published study^34^. The zebrafish was from a transgenic line, Tg(phiC31.attP.2A, − 0.8myl7: EGFP) where the myocardium was tagged with green fluorescence. At 5 days post fertilisation, imaging was performed with a custom-built Line-Scan Focal-Modulation Microscope^35^ in 4D. Focal modulation modulates the fluorescence signal within its focal point and a confocal pinhole that select the modulated signal that come within this focal point. Therefore, reducing the background signal and its interference with the signal from imaging plane. This results in In-plane image resolution of 0.95 $\upmu$m $\times$ 0.90 $\upmu$m. Scans were performed over 120–130 imaging planes, spaced at 1 $\upmu$m. Images at different planes were synchronised with the same method as the chick data to give 4D images with frame rate of 50 frames/s. Images for 3 heart beats were compounded into one by taking its mean.

All animal studies and protocols were approved by the Institutional Animal Care and Use Committee at the National University of Singapore. All research was performed in accordance with relevant guidelines/regulations.

For our data sets, the tracking results are compared against manually segmented geometry which are obtained using VMTK: Vascular Modelling Tool Kit^36^. The measurement of accuracy from our own test cases are shown qualitatively in the volume plot against manually segmented data. Quantitatively, we calculate the Dice coefficient of the tracked geometry against manually segmented images to measure the fit of the volume against manually segmented data. The Dice coefficient is routinely used to quantify the overlap between manual segmented ground truth and automated segmentation in MRI^37–39^.

## Results

### Effect of grid size and Fourier terms

**Figure 2:**
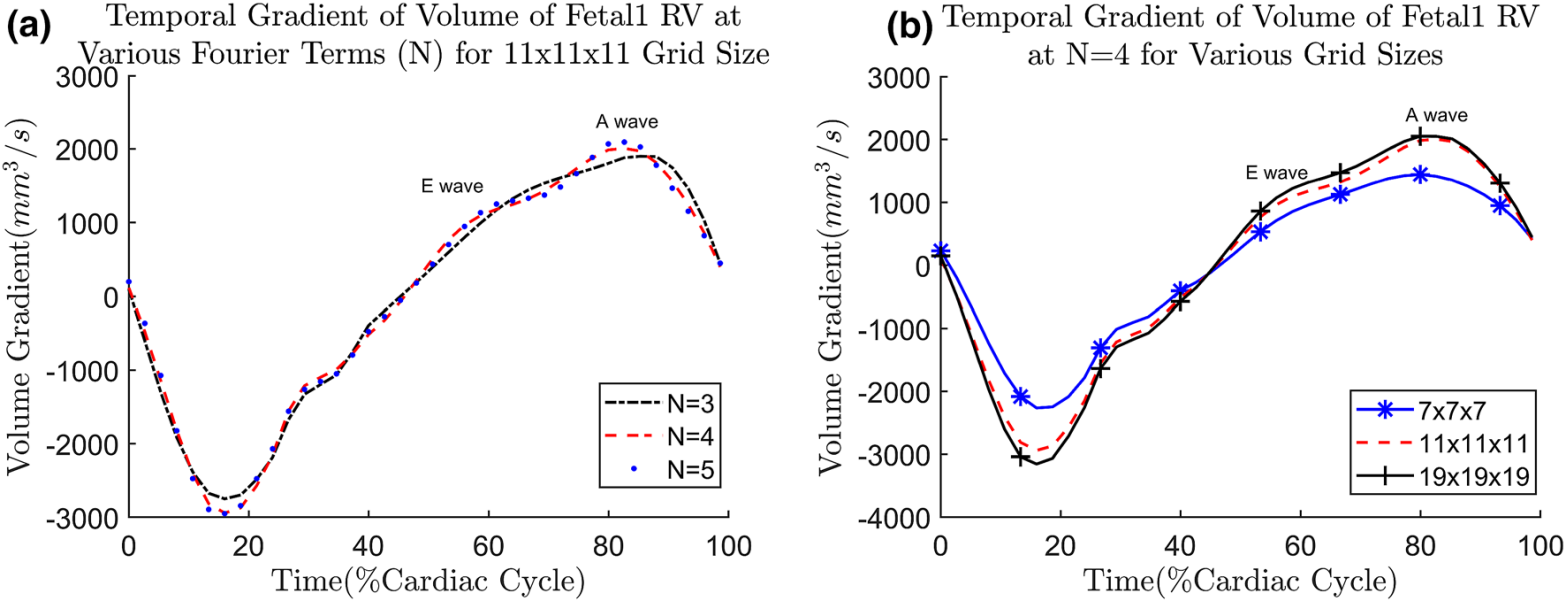
(**a**) Gradient of volume over time curve for the fetal1 RV at various values of *N* at grid size of 11 $\times$ 11 $\times$ 11; (**b**) Gradient of volume over time curve for the fetal1 RV at various grid sizes when $N=4$.

We first demonstrate the effects of different *N* values and b-spline grid size on the Fetal ultrasound cases, the fist few cases available to us. The size of the grid and number of Fourier terms would affect the smoothness of the estimated motion, as shown by the plot of temporal gradient of volume, shown in Fig. 2. The temporal gradient of ventricular volume corresponds to the rate of inflow into or outflow out of the ventricle. It can be observed that having $N<4$ will cause volume gradients corresponding to the E (Elementary) and A (Atrial) waves of ventricular diastolic motion to be dampened out (Fig. 2a). Figure 2a demonstrates that with $N\ge 4$ the volume gradient waveform would show distinct E- and A-waves. Since these dynamic features are considered important indicators of diastolic dysfunction^40^, $N\ge 4$ should be used to maintain these features.

The gradients of volume (i.e. volume flow rate) should follow the shape of inlet/outlet Doppler waveform. Therefore, We perform a frequency analysis of a typical 22 weeks old fetal Doppler inflow/outflow velocity waveform obtained from^41^. We found that the frequency mode of 4 accounts for approximately 96% of the total power and therefore, most of the features in the Doppler. At $N=4$, the E- and A-wave features can indeed be seen. Therefore, we suggest a general cutoff at about 95% of the cumulative power density to reduce noise while keeping these features. This guideline is used across all of the cases studied.

After the value of N is determined, the effect of grid densities are assessed by varying the grid sizes at $N=4$. The multilevel b-spline algorithm used in this study^27^ doubles the amount of control points at each level, with additional of 3 control points at the final level. The grids are tested at various levels: 2, 3 and 4, with a single control point seed at 0th level; resulting in 7 $\times$ 7 $\times$ 7, 11 $\times$ 11 $\times$ 11 and 19 $\times$ 19 $\times$ 19 grids. The volume change over time at various grids is shown in Fig. 2b. We observe that for most of the fetal cases, 11 $\times$ 11 $\times$ 11 at $N=4$ is sufficiently able to capture the E-wave and A-wave. To generalize the choice of grid spacing to the other data sets, the grid spacing and grid size should be related to the size of feature to be tracked. Here, we take the thickness of the myocardium as the feature size, since motion across the thickness of the myocardium should have small variations. For our fetal cases, at the grid size of 11 $\times$ 11 $\times$ 11, the ratio of myocardium thickness to grid spacing is found to be approximately 0.46. This is close to the standard deviation (0.577) of Gaussian kernel approximation to b-spline^42^. Therefore, we ensure the feature length is within one standard deviation of a Gaussian kernel. As an approximate guideline, the ratio of feature length to grid spacing should be close to 0.4–0.6 for all of the cases studied.

### Cardiac motion analysis challenge data

**Figure 3:**
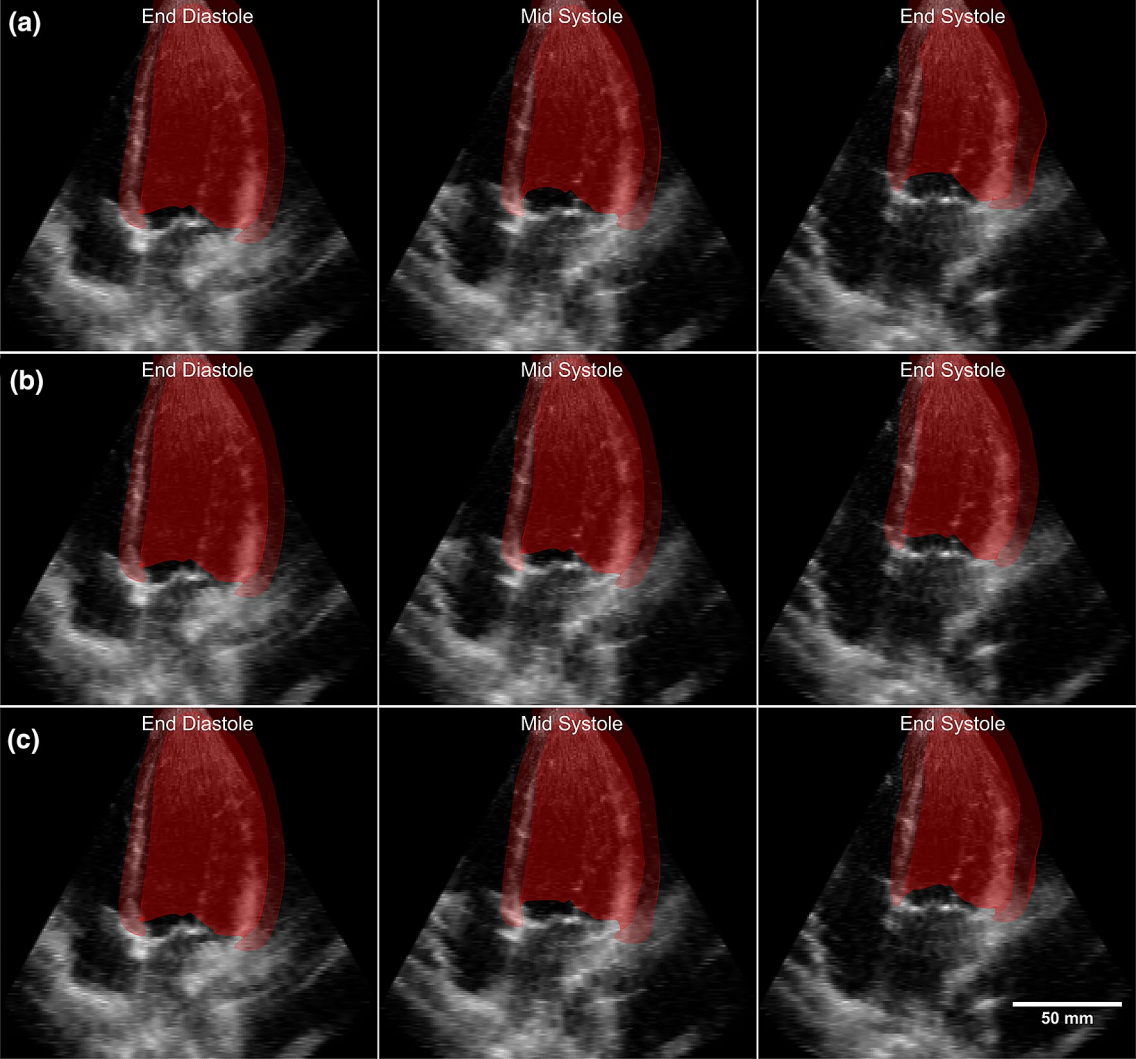
CMAC volunteer 4 tracked geometry from the various steps: (**a**) forward marching (**b**), initialisation (**c**), consistency corrected.

When applied to the cardiac motion analysis data^19^, motion estimation parameters are set to be uniform across all subject cases, with $N=4$, and b-spline grid size of 19x19x19 covering the entire image space. Results from each regularisation step (Forward Marching, Initialisation, and consistency correction) are presented. The results shown in this section are based on the exclusion described in Supplementary Table 2. Results based on the full data is available in the supplementary material (Supplementary Table 3). The fit of the geometry to the image can be seen in Fig. 3 and Supplementary Video 1.

**Figure 4:**
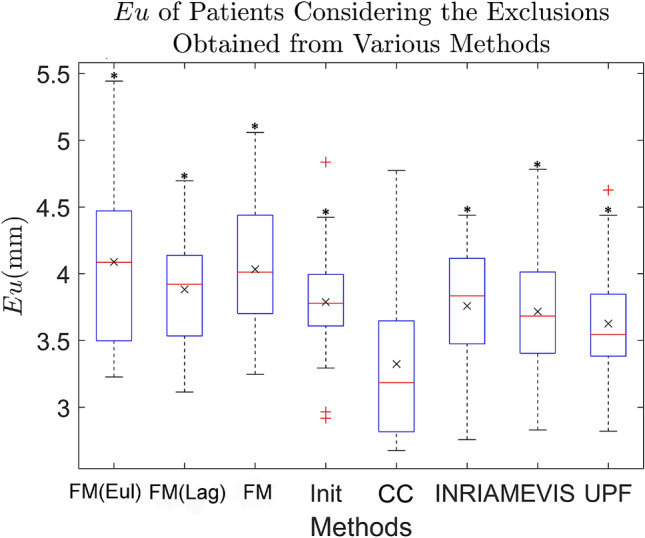
Box plot of the averaged Euclidean distance error (*Eu*) of all patients, for the various methods applied to the CMAC data. The various steps from our proposed framework, using Elastix as the base pairwise registration: *FM* Forward Marching with fully Eulerian (Eul), Lagrangian (Lag) and Quadratic weights, *Init* initialisation, *CC* consistency correction, and the motion estimation algorithms of CMAC participants: *INRIA* Inria Asclepios, *MEVIS* Fraunhofer MEVIS, and *UPF* Universitat Pompeu Fabra. The whiskers are the lowest and highest datum at 1.5 $\times$ of interquartile range assuming normally distributed data. Data beyond the whiskers are considered outliers, labelled with ‘+’. Their average (${\hat{Eu}}$) are labelled as ‘x’. ‘*’ labels p-value $<0.01$ in one-tail paired t-test against CC.

Motion estimation errors are compared to that of the participants of the Challenge. Error for each subject is defined as the temporally averaged Euclidean distance (*Eu*) between manually tracked landmarks to algorithm-tracked landmarks, averaged across tagged all landmarks (LKMS) as shown in equation:

6 $$\begin{aligned} Eu=\frac{\sum \limits ^{n_{LMKS}}\sum _{t=0}^{P}||\vec {X}^t-\vec {X}_{Truth}^t||}{n_{LMKS}\times P} \end{aligned}$$

where $\vec {X}_{Truth}^t$ is the ground truth physical Cartesian coordinate of a landmark as provided by the Challenge and $n_{LMKS}$ is the number of elements within LMKS used. The comparison of methods’ accuracy by means of Euclidean distance is done similarly in CMAC^19^. The motion estimation errors (*Eu*) averaged for all subjects, represented by ${\hat{Eu}}$, is also shown in Fig. 4.

Results from Elastix registration demonstrate that every regularisation step improves on the previous. The error from simple marching methods such as Eulerian (4.09 ± 0.65 mm), Lagrangian (3.88 ± 0.44 mm) and forward marching (3.97 ± 0.45 mm) is often higher than the CMAC participants. When regularisation is performed, at the initialisation step, tracking errors are comparable with results from CMAC participants, with an ${\hat{Eu}}$ of 3.80 ± 0.42 mm (mean ± standard deviation). With the consistency correction, ${\hat{Eu}}$ improved to 3.32 ± 0.54 mm. This is 10% lower than errors achieved by participants of the Challenge: INRIA = 3.69 ± 0.44 mm, MEVIS=3.74 ± 0.53 mm, UPF = 3.81 ± 0.45 mm. Paired one-tailed t-test between the consistency correction results against any other group shows significant difference with p-values $<0.0004$. Without excluding any data, the improvement is consistent with p-values of $<0.0404$. Using SLDD registration, results in forward marching error of 4.61 ± 0.43 mm, initialisation error of 4.50 ± 0.41 mm, and consistency correction error of 4.15 ± 0.44 mm. However the consistency correction errors are still higher than participants of the challenge. This is likely due to high error already present at the forward marching step. For this reason we would apply only Elastix registration on the rest of the data sets.

### Human fetal ultrasound data

Human fetal cardiac ultrasound data generally have high noise, due to the depth of imaged object, small size of the fetal heart (approximately 1cm in diameter at 21st weeks of gestation), and shadowing by fetal bones. This set of data thus serves as good litmus test on the robustness of our proposed framework. For fetal data sets, $N=4$ and b-spline grid size = 11 $\times$ 11 $\times$ 11 were used. We perform motion estimation for the right ventricle (RV).

**Figure 5:**
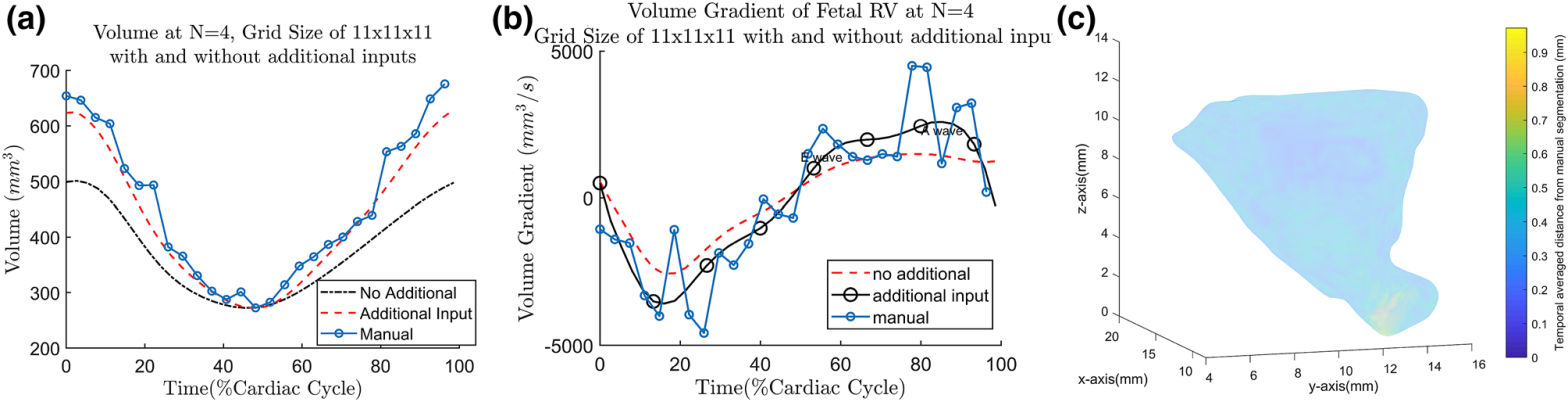
(**a**) Volume of the fetal1 RV obtained from manual segmentation compared to the proposed method, with and without additional inputs of the additional displacement field from the end diastolic to end systolic time frames; (**b**) temporal gradient of volume over time curve for the same set of data; (**c**) surface contour depicting temporally averaged Euclidean distance error between the motion estimated mesh and the manually segmented mesh.

To gauge the accuracy of our motion estimation, computed surfaces are compared to those from manual segmentation. Figure 5 shows the plot of fetal1 RV volume over time, and demonstrates that the consistency correction result is in phase with manually segmented volume, but the RV stroke volume (difference between the maximum and minimum volume) is underestimated. The further step of additional input vectors, is found to be able to rectify this, resulting in a better fit. In this implementation, the additional displacement field is obtained from registering the segmented image frames at which the RV is at its end diastole and systole volumes. This field is given a weight of 10 times that of other image registrations. This additional input does not affect the shape of the volume over time waveform, but ensures that the waveform’s magnitude fits the constraint imposed by the additional input. Compared to Forward Marching alone, this Additional Input step demonstrated a decrease in stroke volume errors from 20.2 ± 2.5% to 12.0 ± 0.5%. The Additional Input step of our regularization add-on layers can thus be a good way to reduce stroke volume errors from registration results.

**Figure 6:**
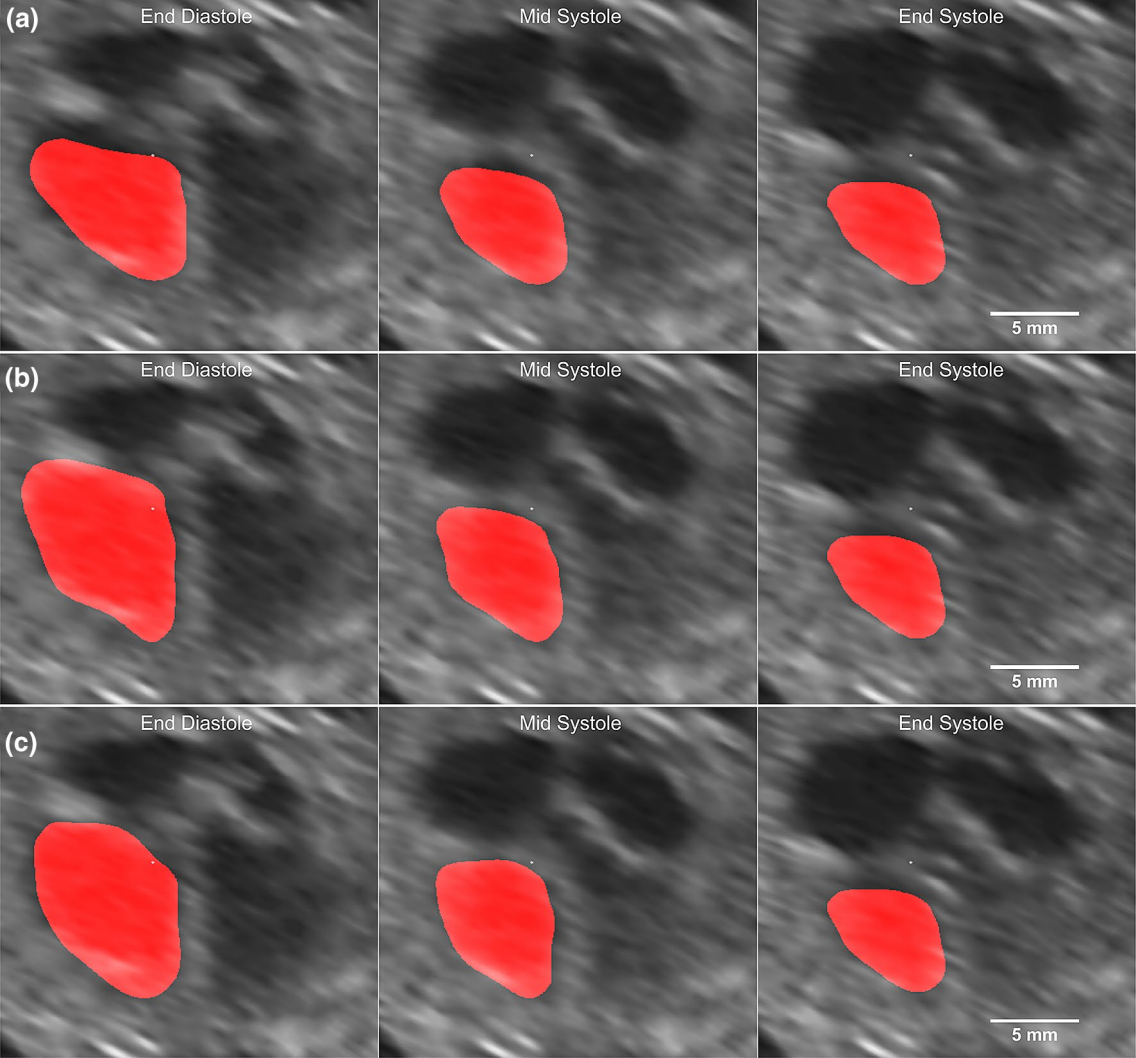
Fetal1 right ventricle geometry overlaid on top of ultrasound at different time points for (**a**) consistency corrected data without additional input, (**b**) consistency corrected data with additional vector inputs, (**c**) manually segmented geometry.

To illustrate the spatial distribution of the fit between our motion estimated surfaces and manually segmented ones, the temporal averaged Euclidean distance errors are plotted as color contours on the surface, as shown in Fig. 5c. The Euclidean distance error, averaged over the three cases, is: 0.56 ± 0.10 for forward marching and improved to 0.43 ± 0.11 mm after additional inputs (Table 1). This is approximately 5% of the width of the RV. In this plot, it can be observed that the inlet and outlet region has the largest errors. This is because at these openings, the ultrasound imaging cannot capture distinct boundaries for motion estimation. The valves at these locations can be seen on some frames, but their dynamic motions are too fast and appear as blurs. Figure 6, with Supplementary Videos 2 and 3, shows plots of the 3D motion estimated surfaces on top of ultrasound images to qualitatively demonstrate the good fit.

**Table 1:** Euclidean distance error and DICE coefficient against manual segmentation for our dataset. *FM* Forward marching, *Init* initialisation, *CC* consistency correction, *Add* additional inputs.

Case	Metric	Eu	DICE
Fetal	FM	0.56 ± 0.10 mm	0.826 ± 0.059
	Init	0.48 ± 0.13 mm	0.827 ± 0.045
	CC	0.45 ± 0.12 mm	0.829 ± 0.092
	Add	0.43 ± 0.11 mm	0.849 ± 0.091
Zebrafish	FM	4.74 ± 0.46 µm	0.812 ± 0.062
	Init	4.42 ± 0.63 µm	0.834 ± 0.049
	CC	4.34 ± 1.24 µm	0.847 ± 0.057
Chick	FM	0.034 ± 0.006 mm	0.887 ± 0.023
	Init	0.029 ± 0.006 mm	0.892 ± 0.027
	CC	0.028 ± 0.004 mm	0.898 ± 0.023
	Add	0.030 ± 0.005 mm	0.898 ± 0.030

Value is presented as mean ± standard deviation of used metric over the three cases.

To further gauge accuracy, the Dice coefficients are calculated, averaged over time and all the three cases studied. Forward marching Dice coefficient is 0.826 ± 0.059 and after additional vector input to be 0.849 ± 0.091 (Table 1).

### Embryonic hearts from confocal microscopy and high-frequency ultrasound data

**Figure 7:**
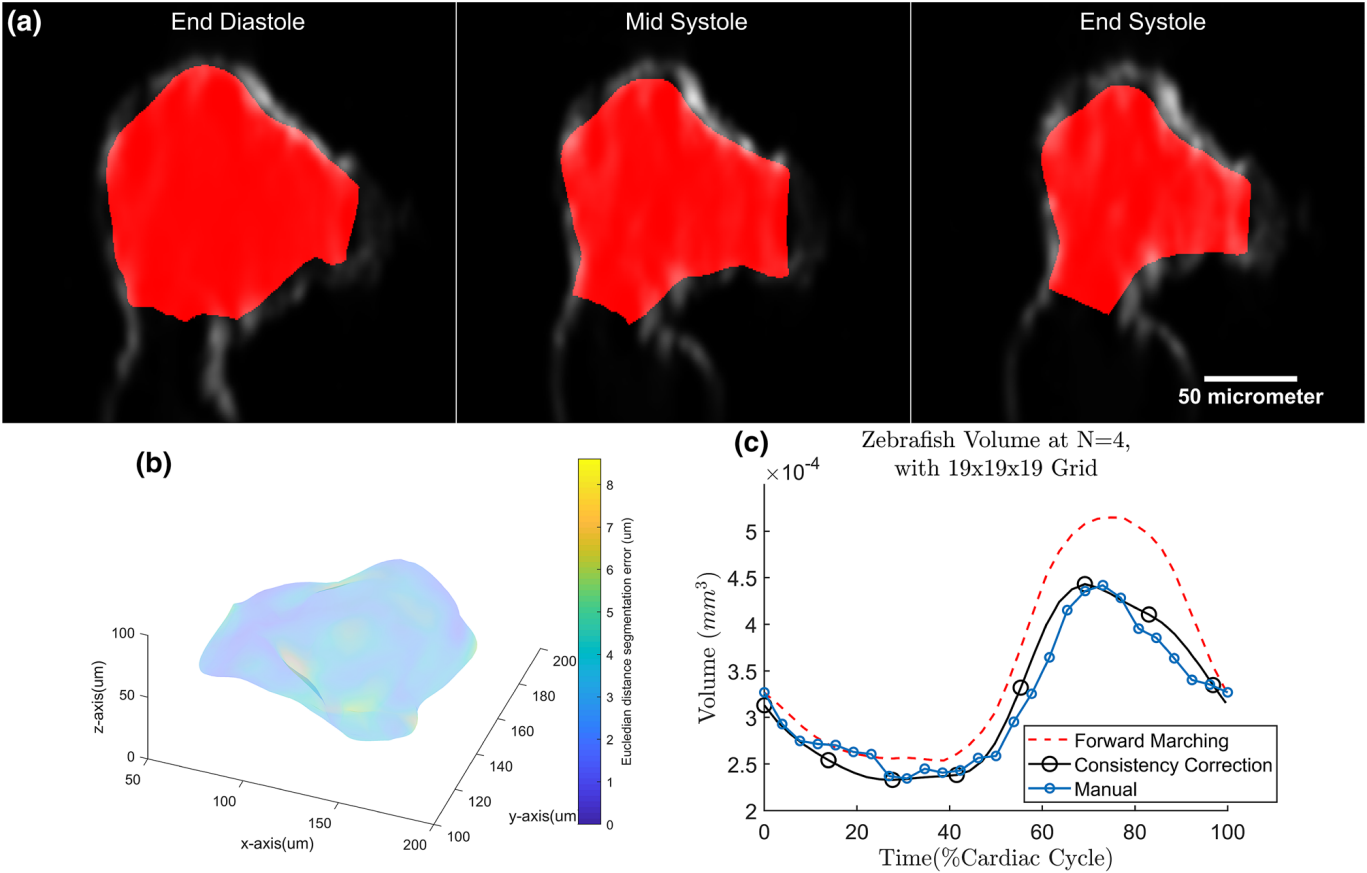
(**a**) Motion estimated zebrafish1 ventricle on top of its confocal microscope image at different time points. (**b**)Temporal averaged Euclidean distance error between motion estimated surface and manually segmented surface of zebrafish1 ventricle. The surface color contour represents the magnitude of Euclidean distance error; (**c**) volume changes of zebrafish1 ventricle.

In the zebrafish embryonic heart data sets, ventricular motion estimation are performed until the consistency correction step with grid size of 19 $\times$ 19 $\times$ 19 and $N=4$. Qualitatively, good fit can be gauged from the overlay of the 3D tracked surfaces as well as the confocal images in Fig. 7a and Supplementary Videos 4 and 5. The fit of volume over time is also shown in Fig. 7c. Averaged across the three cases, the Dice coefficient of the forward marching is found to be 0.812 ± 0.062, improves to 0.847 ± 0.057 after consistency correction (Table 1). Locally, the goodness of fit can be quantified as the Euclidean distance error (Fig. 7b). From Fig. 7b, Euclidean error is small everywhere, and has a surface average over the three cases as 4.74 ± 0.46 at forward marching and decreased to 4.32 ± 1.24 $\upmu$m after consistency correction (Table 1). This error is approximately 6% of the heart’s width.

**Figure 8:**
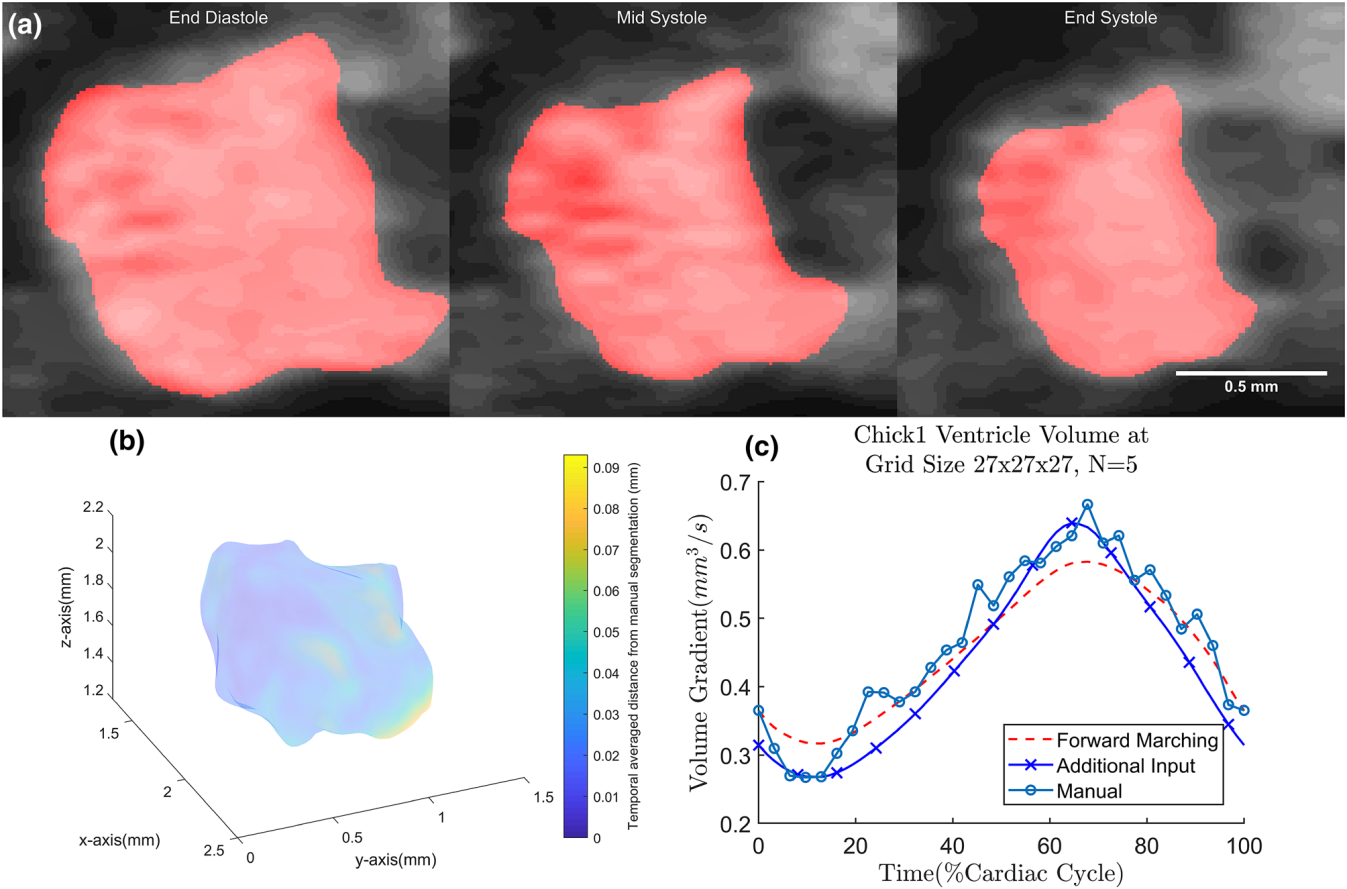
(**a**) Chick1 embryo ventricle geometry, obtained from Additional Input, overlaid on top of its ultrasound image at different time points; (**b**) temporal averaged Euclidean distance difference between the mesh obtained from the proposed framework and manually segmented mesh for chick1 ventricle. The surface color contour relates to the magnitude of Euclidean distance error between motion estimated surface and manual segmented surface; (**c**) volume over time curve for the chick1 ventricle.

In the chick embryonic heart high-frequency ultrasound data sets, ventricular motion estimation is performed until the consistency correction step with b-spline grid size of 27 $\times$ 27 $\times$ 27 at $N=5$. The quality of fit can be demonstrated with the overlay of the 3D tracked ventricle on the ultrasound images in Fig. 8a and Supplementary Videos 6 and 7. The fit of its volume over time can also be seen in Fig. 8c. Averaged across the three cases, the Dice coefficient of the forward marching is found to be 0.887 ± 0.023, improves to 0.898 ± 0.030 after consistency correction (Table 1). Locally, the distribution of error can be observed in Fig. 8b as Euclidean distance error. The surface averaged Euclidean distance error averaged across the three cases at forward marching is 0.034±0.006 and reduced to 0.028 ± 0.004 mm after consistency correction (Table 1). Approximately 6.5% of the ventricle’s width. With Additional Inputs, stroke volume errors are observed to decrease from 26.6 ± 7.3% after Forward Marching to 8.2 ± 1.1%.

## Discussion

In the current study, we propose an add-on regularisation layer on pairwise image registration, a novel motion model is developed and tested on various data set. This add on layer was tested on two different registration methods: Elastix and SLDD. Improvement was observed in both methods, but more so with Elastix, reducing its error to be lower than participants of CMAC by approximately 10%. We thus believe that an add-on regularisation layer is viable, and showed that it can lead to improved accuracy.

On top of accuracy, the regularisation framework enforced smoothness, cyclicity of cardiac motions, and temporal consistency, due to the use of the global geometric transformation model based on BSF. Fourier and b-splines functions were used as they are well understood, continuous and easily differentiable. These mathematical forms also naturally enforce smoothness, which can be controlled with the choices on grid sizes and number of Fourier terms. Additionally, the Fourier formulation guarantees cyclic motion without needing to penalise drift in the objective function during registration. Smoothness and cyclicity can thus be enforced without the need for specific regularisation terms in the objective function, which can complicate the optimisation process during registration.

The use of our global temporal modelling leads to improved accuracy, as observed from error reduction in subsequent steps. The model takes into account the information from other time points, which may improve tracking accuracy. Further, the simultaneous curve-fitting of the model onto all time points and across space allowed bridging of localized noise and signal losses. In terms of temporal consistency, since motion at any point is described with a single equation covering all time points, temporal consistency is ensured. However, the use of cyclic motion as a prior would not be able to account for all medical conditions. Particularly in patients with cardiac abnormality that violate the cyclic assumption made such as arrhythmia.

From our investigation, the consistency correction step is important. In both registration approaches tested, it produces the most significant improvement in accuracy (Fig. 4). The consistency correction step also have the important flexibility of allowing any set of registration displacement fields to be used as inputs. In the fetal and chick embryo heart data, we use Additional Input Vectors and demonstrate that the estimated motion has improved adherence to the stroke volumes from manually segmented ground truth as shown by the reduced stroke volume errors. During image registration, smoothing regularisation may lead to underestimation of the stroke volume. Further, in fetal ultrasound, accurate tracking of the stroke volume can be difficult. The relaxation of the trabeculations during diastole leads to the filling of blood volumes within the interstitial trabeculation spaces. This caused the trabecula signals to appear dark, and their subsequent contraction during systole can led to the same signal appearing bright as the fluid were pushed out of the spaces. This suggest a shift in the tracking objective: at end diastole, the inner surface of the myocardium should delineate the volume of blood in the ventricle, but at end systole the trabecular surface should be the new boundary. The additional input allows smooth transition of the tracked boundary from the myocardium to the trabecula. This highlights the utility of the Additional Input flexibility of our framework.

However, our method has the limitation of dependency on the pairwise registration results. Registration results are assumed to be accurate, such that deviation from this vector fields are minimised. This dependency can be demonstrated by comparing SLDD and Elastix results. Consistency Correction processing provided more accurate results when applied onto the more accurate registration output. The pairwise registration accuracy are dependent on the registration parameters such as the choice of similarity and regularisation function as well as their corresponding weights, and on the quality of the images. However, in our current work, optimisation of these parameter are not comprehensively conducted (some are demonstrated in Supplementary Table 5) and should not be taken as the conclusive comparison between various registration methods. Nonetheless, our proposed regularisation add-on framework can still demonstrate an improvement in tracking results regardless of which registration algorithms is tested, whether SLDD or Elastix (Supplementary Table 4).

Another issue of concern is the diffeomorphism and incompressibility constraint. Diffeomorphism constraint dictates that the relative location of the tissues remained unchanged after transformation, while incompressibility dictates that tissues maintain its volume after the transformation. Mathematically, diffeomorphism is defined with det(Jacobian) of transformation is more than 0 and incompressibility as det(Jacobian) = 1. These two constraints are not accounted for in this particular study. However, our transformation’s Jacobian is checked within the myocardium mesh given from CMAC. We find only three out of fifteen patients having negative value for the minimum det(Jacobian). However, the average det(Jacobian) are close to 1 (Supplementary Table 6). Among the three cases, only a maximum of 4 points within the mesh have a negative det(Jacobian). Further investigation showed that this is caused by the myocardium segmentation in CMAC (obtained from tagged MRI) being misaligned with the myocardium space in ultrasound images. After re-segmentation of the myocardium from the ultrasound images and repeating the motion estimation process, negative det(Jacobian) is no longer observed. As a further step to ensure the diffeomorphic constraint, we propose the possibility of adding another regularisation layer after the consistency correction step, by enforcing incompressibility, increasing deformation velocity field smoothness or constraining det(Jacobian) values.

## Supplementary information

Supplementary Information.

Supplementary Video 1.

Supplementary Video 2.

Supplementary Video 3.

Supplementary Video 4.

Supplementary Video 5.

Supplementary Video 6.

Supplementary Video 7.
