# Supplementary material for: Cardiac motion estimation from medical images: a regularisation framework applied on pairwise image registration displacement fields
Source: Sci Rep. 2020 Oct 28;10:18510. doi: 10.1038/s41598-020-75525-4 (PMC7595231; doi:10.1038/s41598-020-75525-4)
Supplement: Supplementary file 1 — Supplementary Information. [file 41598_2020_75525_MOESM1_ESM.pdf]

Cardiac Motion Estimation from Noisy Medical Images: A Regularisation Framework Applied on Pairwise Image Registration Displacement Fields

Wiputra Hadi[1,+], Chan Wei Xuan[1,+], Foo Yoke Yin[1], Ho Sheldon[1], and Yap Choon Hwai[1,*]

[1]National University of Singapore, Department of Biomedical Engineering, Singapore, 117583
[2]Imperial College London, Department of Bioengineering, London SW7 2AZ
[*]c.yap@imperial.ac.uk
[+]these authors contributed equally to this work

1 Supplementary Material

1.1 Various Forward Marching Weights

Supplementary Table 1. Average Euclidean Error of CMAC data (with exclusion) using various Lagrangian and Eulerian weights

Weights	Quadratic	Gaussian($\sigma = \frac{P}{4}, \mu = \frac{P}{2}$)	Full Lagrangian
Initialisation	3.79±0.42	3.98±0.45	3.80±0.38
Consistency Correction	3.32±0.54	3.32±0.54	3.32±0.54

1.2 Exclusion Criteria

Supplementary Table 2. Exclusion table of the two observers (OBS) for the different Volunteers (V).

OBS1	V1	V2	V4	V5	V6	V7	V8	V9	V10	V11	V12	V13	V14	V15	V16
LMKS1	0	0	0	0	0	1	1	0	0	1	0	0	0	0	0
LMKS2	0	0	0	0	0	1	1	0	0	1	0	0	0	0	0
LMKS3	0	0	0	0	0	1	1	0	0	1	0	1	0	0	0
LMKS4	0	0	0	0	0	1	1	1	0	0	0	1	0	0	0
LMKS5	0	0	0	0	0	1	1	0	0	1	0	1	0	0	0
LMKS6	0	0	0	0	0	1	1	0	0	1	0	1	0	0	0
LMKS7	0	0	0	0	0	1	1	0	0	1	0	1	0	0	0
LMKS8	0	0	0	0	0	1	1	0	0	1	0	1	0	0	0
LMKS9	0	0	0	0	0	1	1	0	0	1	0	0	0	0	0
LMKS10	0	0	0	0	0	1	1	0	0	1	0	1	0	0	0
LMKS11	0	0	0	0	0	1	1	0	0	1	0	1	0	0	1
LMKS12	0	0	0	0	0	1	1	0	1	1	0	0	0	0	0
OBS2	V1	V2	V4	V5	V6	V7	V8	V9	V10	V11	V12	V13	V14	V15	V16
LMKS1	0	0	0	0	0	1	1	0	0	1	0	0	0	0	1
LMKS2	0	0	0	0	0	1	1	0	0	1	0	0	0	0	0
LMKS3	0	0	0	0	0	1	1	0	0	1	0	0	0	0	0
LMKS4	0	0	0	0	0	1	1	0	0	0	0	0	0	0	0
LMKS5	0	0	0	0	0	1	1	0	0	1	0	0	0	0	0
LMKS6	0	0	0	0	0	1	1	0	0	1	0	0	0	0	0
LMKS7	0	0	0	0	0	1	1	0	0	1	0	0	0	0	0
LMKS8	0	0	0	0	0	1	1	0	0	1	0	0	0	0	0
LMKS9	0	0	0	0	0	1	1	0	0	1	0	0	0	0	0
LMKS10	0	0	0	0	0	1	1	0	0	1	0	0	0	0	0
LMKS11	0	0	0	0	0	1	1	0	0	1	0	0	0	0	0
LMKS12	0	0	0	0	0	1	1	0	0	1	0	0	0	0	0

Data labelled with '1' suggest exclusion due to repeats of more than 3 coordinates consecutively over time. LMKS are the landmarks being tagged.

Exclusion table for cardiac motion challenge data is demonstrated in table 2. Since Volunteer 7,8 and 11 (V7,8,11) have more than 50% of the landmarks excluded, therefore the entire volunteers' data would be excluded. Observer 1 data of V13 would be excluded for the same reason, while observer 2 of V13 data is still acceptable.

1.3 Consistency Correction's Gradient Descent

The initial value of $\vec{F}^*$ is obtained using $\vec{C}^{init}$ values, and updated based on the following formulation:

$$\vec{\mathscr{F}}_{n+1} = \vec{\mathscr{F}}_n + \beta \vec{d}_n + (1-\beta)\vec{d}_{n-1}$$
$$\vec{d}_n = [H + \lambda I]^{-1} d\vec{i}f_n \nabla d\vec{i}f_n$$
$$H = (\nabla d\vec{i}f_n)(\nabla d\vec{i}f_n)^T \tag{1}$$

Where Where $\vec{\mathscr{F}}_n$ includes $\vec{F}$ at all frequency modes, rearranged as a vector. $\vec{d}_n$ is the descent direction at iteration n, H is the pseudo-Hessian matrix, λ is the damping parameter, initialised with $\lambda = 0.001$ and updated according to[1] with an update factor of 5. (1-β) is the weight of the momentum term that further dampens the change in $\vec{d}$ based on $\vec{d}_{n-1}$.

Convergence is achieved when the change in $\vec{\mathscr{F}}_n$ over an iteration is small, where $\varepsilon = 10^{-5}$:

$$|\vec{\mathscr{F}}_n - \vec{\mathscr{F}}_{n-1}| < \begin{cases} \varepsilon|\vec{\mathscr{F}}_n| & \text{, when } |\vec{\mathscr{F}}_n| \neq 0 \\ \varepsilon \times min(a,b,c) & \text{, when } |\vec{\mathscr{F}}_n| = 0 \end{cases} \tag{2}$$

1.4 Lagrangian Tracking Gradient Descent

$\vec{X}_{ref}$ will descent from its known coordinate at time t. This value is updated based on formulation in equation (1), but with $\vec{\mathscr{F}}$ replaced by $\vec{X}_{ref}$. λ is kept to $10^{-5} < \lambda < 1$ with an initial value of 0.01 and an update factor of 10, β set to be 0.8, and $\vec{d}_{n-1}$ is set to zeros when $n = 1$.

During the descent, a measure of relative descent speed is used to detect local minima, plateau and oscillations, which we found to be quite common. The relative descent speed ($Cost^n_{rel}$) is defined as the absolute change in exponential moving average cost function ($Cost^{n-1}_{ave}$) over the iteration, normalised against the last iteration's averaged cost:

$$Cost^n_{ave} = \frac{Cost^{n-1}_{ave} + Cost^n}{2}$$
$$Cost^n_{rel} = \frac{|Cost^n_{ave} - Cost^{n-1}_{ave}|}{Cost^{n-1}_{ave}} \tag{3}$$

At the start of the iteration, $Cost^0_{ave}$ can be set to any large number, such as 100. When a point is stuck at a local minima or plateau $Cost^n_{rel}$ will be low due to diminishing gradient. $Cost^n_{rel}$ is also designed to detect oscillations since $Cost^n_{ave}$ remained relatively constant under oscillation. We detect these situations with the criteria that $Cost^n_{rel} < 0.4$, and if found, a new starting point is randomised within ± 2 b-spline grid spacing from the old starting point and the descent is repeated. This imparts a slight stochastic property into the algorithm. Convergence is considered achieved when $Cost(\vec{X}_{ref}) < 10^{-6}$, which took less than 50 iterations in most cases.

1.5 CMAC Result without Exclusion

Supplementary Table 3. Averaged Euclidean distance error $\hat{Eu}$ on all cardiac motion analysis data for various methods, using Elastix as a base pairwise registration: FM - Forward Marching, Init - Initialisation, CC - Consistency Correction, INRIA - Inria-Asclepios, MEVIS - Fraunhofer MEVIS, and UPF - Universitat Pompeu Fabra

Methods	FM	init	CC	INRIA	MEVIS	UPF
$\hat{Eu}(mm)^*$	4.22 ± 0.65	3.87 ± 0.45	3.47 ± 0.63	3.69 ± 0.40	3.74 ± 0.57	3.81 ± 0.79
p-value**	4.1×10^{-11}	3.3×10^{-6}	-	3.6×10^{-2}	2.9×10^{-3}	2.1×10^{-4}

* values as mean $\pm$ standard deviation

** p-values are obtained from one-sided paired t-test of various methods against CC

1.6 $\hat{Eu}$ of Symmetric Log Domain Demons (SLDD) and Free Form Deformation (FFD) Registration

We calculated average Euclidean distance errors of the simple Eulerian and Lagrangian marching without our regularization, and errors after our regularization (the BSF Consistency Correction). Table 1 below shows the results, demonstrating that our proposed regularization framework could reduce errors ($\hat{Eu}$) from both registration methods, regardless of their initial accuracies.

Further, optimisation of the demon's result were performed by varying Gaussian standard deviation were tested, with (SLDD) or without (LDD) symmetric forces and cost function. Whichever method, Consistency Correction showed reduction in Euclidean error.

Supplementary Table 4. Average Euclidean distance errors of simple marching of FFD and LDD registrations, and those after our proposed regularization framework.

Method	FFD Registration			LDD Registration		
	Eulerian	Lagrangian	Consistency Correction	Eulerian	Lagrangian	Consistency Correction
$\hat{Eu}$	4.09	3.88	3.32	4.10	4.47	4.01

Supplementary Table 5. Average Euclidean distance errors of Demons registrations. FM- Forward Marching, Init-Initialisation, CC- Consistency Correction

Method	SLDD			LDD		
	FM	Init	Consistency Correction	FM	Init	Consistency Correction
Gaussian=0px	6.06±0.74	5.09±0.52	4.84±0.47	6.82±0.77	5.50±0.75	4.83±0.49
Gaussian=1.5px	4.61±0.43	4.50±0.41	4.15±0.44	4.97±0.48	4.81±0.50	4.15±0.43
Gaussian=3px	6.82±0.77	5.50±0.75	4.83±0.49	4.48±0.45	4.48±0.47	4.01±0.45

1.7 Jacobian of the Transformation

From the CMAC data, the segmentation of myocardium were already given. We used the segmentation as a mask on the image. The Jacobian were obtained at points that were sampled at regular interval of 1/4 the width of the voxel in all 3 dimensions within the myocardium. This were repeated for 40 regularly spaced time points per cardiac cycle.

Supplementary Table 6. Jacobian of transformation across all CMAC patients

Patients	V1	V2	V4	V5	V6	V7	V8	V9	V10	V11	V12	V13	V14	V15	V16
Minimum Jacobian	0.10	0.16	0.24	<0	0.36	<0	0.14	0.21	0.19	0.14	0.02	<0	0.11	0.26	0.07
Average Jacobian	1.00	0.96	0.95	0.99	1.02	0.93	0.94	0.92	0.95	0.97	0.94	0.97	0.98	0.98	1.02

References

1. Roweis, S. Levenberg-marquardt optimization. *Notes, Univ. Of Tor.* (1996).
